# Synergistic mode of action of a ceftazidime-hexa-arginine conjugate with resistance-overcoming properties

**DOI:** 10.1128/spectrum.00693-26

**Published:** 2026-07-13

**Authors:** Hannah Isabell Müller, Julia Werner, Julia Guther, Jan Straetener, Manuel Ben Böhmann, Philipp Uhl, Heike Brötz-Oesterhelt

**Affiliations:** 1 Microbial Bioactive Compounds, Interfaculty Institute of Microbiology and Infection Medicine, University of Tübingen 9188, Tübingen, Germany; 2 Department of Nuclear Medicine, University Hospital Heidelberg 27178, Heidelberg, Germany; 3 Institute of Medical Microbiology and Hygiene, University Hospital Tübingen 27203 , Tübingen, Germany; 4 Department of Pharmaceutical Technology, Institute of Pharmacy and Molecular Biotechnology, University of Heidelberg, Heidelberg, Germany; 5 Cluster of Excellence Controlling Microbes to Fight Infection, Tübingen, Germany; 6 German Center for Infection Research, Partner Site Tübingen, Tübingen, Germany; Ludwig-Maximilians-Universitat Munchen Pettenkofer Institute, München, Germany

**Keywords:** antibiotic discovery, antibiotic multi-resistance, ceftazidime, β-lactam, polycationic peptide, conjugate, mode of action

## Abstract

We previously described that a non-toxic ceftazidime-hexa-arginine conjugate (CTZ-R6) surpassed the antibacterial potency of ceftazidime (CTZ) against multi-resistant bacteria by up to 1,000-fold and effectively reduced the bacterial load in murine bacteremia. However, the mechanistic understanding of its resistance-overcoming activity remained elusive. Here, we report on the mode of action of CTZ-R6 in gram-positive and gram-negative bacteria. Against a panel of multi-resistant Enterobacteriaceae and non-fermenters, CTZ-R6 outperformed ceftazidime. The conjugate synergized with avibactam in the same manner as CTZ, but its MICs were much lower than those of CTZ. CTZ-R6 crossed the outer membrane of *Escherichia coli* by self-promoted uptake and permeabilized this barrier to a fluorescent dye and otherwise ineffective antibiotics. Investigations in *Bacillus subtilis* demonstrated that the electrochemical gradient at the cytoplasmic membrane remained high, but membrane topology was perturbed, and the peptidoglycan sacculus was damaged. Lysis kinetics were β-lactam-like. CTZ-R6 showed higher affinity than CTZ for penicillin-binding protein 2a (PBP2a) of *B. subtilis*, lower affinity for PBP1a/b, and an overall more balanced PBP affinity than CTZ. The covalent linkage between CTZ and the hexa-arginine moiety was essential for potency. In dissecting the contributions of the individual moieties, we found that R6C destabilizes the outer membrane, enabling porin-independent uptake. The peptide also interacts with the cytoplasmic membrane, facilitating CTZ access to the PBPs in *B. subtilis*. More balanced targeting of PBPs and porin-independent entry is beneficial for overcoming established resistances. In conclusion, both components of the conjugate contribute to the increased antibacterial potency of CTZ-R6 in a balanced and synergistic manner.

**IMPORTANCE:**

Given the global scale of antibacterial resistance, it becomes clear that numerous infections may eventually become untreatable as former, well-established treatment regimens fail. To prevent the emergence of a post-antibiotic era, strategies are being explored to extend the usefulness of clinically important antibiotics, including the synthesis of conjugates that improve the potency of established drugs. We recently demonstrated that attaching polycationic peptides to all four β-lactam subclasses improved MICs against multi-resistant gram-positive and gram-negative pathogens. The ceftazidime-hexa-arginine conjugate showed the best overall profile and improved the potency of ceftazidime against multi-resistant bacteria by up to 1,000-fold. However, the molecular mechanism of its resistance-overcoming activity remained elusive. Here, we describe which characteristics and capabilities of the peptide and the β-lactam allow them to synergize for increased potency. Mechanistic understanding is essential for further development of such conjugation approaches.

## INTRODUCTION

Antibiotic resistance is one of the most pressing current health threats worldwide (1). Due to its steady increase, numerous bacterial infections may eventually become untreatable, resulting in soaring mortality rates and public health costs (2, 3). Beyond infection treatment, many other medical interventions are at risk if well-established antibiotic treatment regimens fail (4).

β-lactams are the most widely used class of antibiotics (5). Well-tolerated and efficacious, they account for about 65% of the global antibiotic market (6). Despite the wide range of β-lactam derivatives available (5) with different pathogen spectra, as well as pharmacological and pharmacokinetic benefits, they all share the same molecular mechanism (5). The β-lactam ring is a structural analog of the terminal amide bond in the peptidoglycan precursor, and enzymes processing this bond (i.e., penicillin-binding proteins [PBPs]) are covalently inactivated by β-lactam antibiotics. Impaired peptidoglycan cross-linking results in an aberrant cell wall structure (7). The presence of a β-lactam ring makes β-lactam antibiotics susceptible to β-lactamases, a diverse group of bacterial enzymes that hydrolyze the ring and thereby confer β-lactam resistance (8). This resistance mechanism can be mitigated by combining β-lactam antibiotics with β-lactamase inhibitors (6, 9).

Cephalosporins are often prescribed β-lactam antibiotics (5). Ceftazidime (CTZ), a broad-spectrum, third-generation cephalosporin, is used to treat infections of the skin and respiratory or urinary tract, caused by both gram-positive and gram-negative bacteria, e.g., *Pseudomonas aeruginosa* and Enterobacteriaceae (10). In fixed combination with the β-lactamase inhibitor avibactam (AVI) (11), CTZ represents an important line of defense against multi-resistant gram-negative pathogens of global health concern. However, resistance to the CTZ/AVI combination is also rising (12). In general, besides (hyper)production of β-lactamases, β-lactam resistance occurs as a result of overexpression of efflux pumps (13), loss of porins or their mutation (14), or modifications in PBPs (15).

To extend the lifespan of β-lactams and other important antibiotics, innovative, resistance-overcoming approaches are essential. Recently, we reported that the potency of cell-wall targeting antibiotics can be substantially increased by fusing them to polycationic peptides. Using vancomycin as a first model antibiotic, we explored various peptides, linkers, fatty acid extensions, and pegylation. Conjugates containing six linear arginine residues turned out particularly efficacious (16, 17). The hexa-arginine moiety proved promising when fused to representatives of all four β-lactam subclasses, and particularly, high efficacy was observed for a conjugate with CTZ (18).

**Fig 1:**
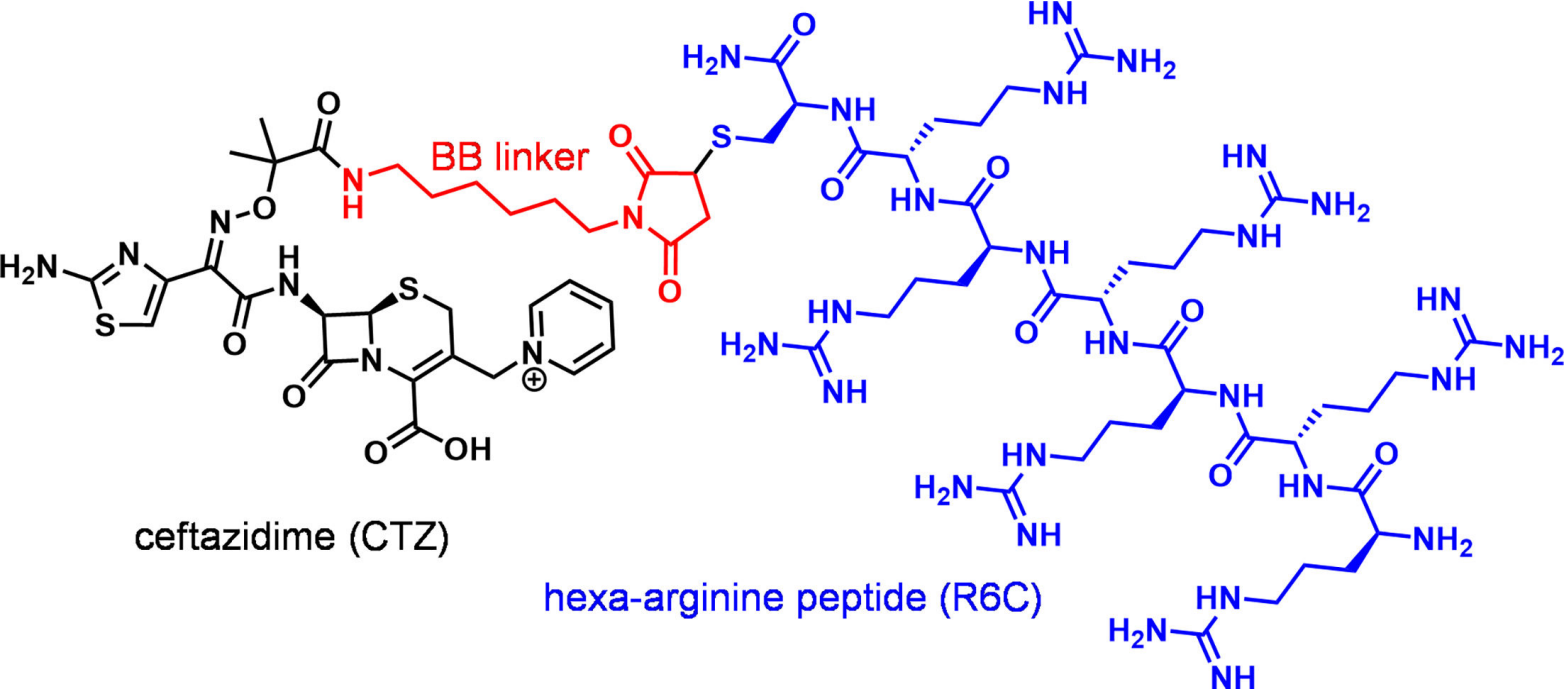
Structure of the conjugate CTZ-R6 containing CTZ, R6C, and a BB (6-maleinimidohexanamide) linker.

Conjugate CTZ-R6 (Fig. 1) was prepared by joining CTZ to the heterobifunctional linker 6-maleinimidohexanamide (BB linker) using a position that did not interfere with the activity of the β-lactam ring, followed by conjugating the linker to the hexa-arginine peptide R6C via a connecting cysteine (18). The potency of CTZ-R6 surpassed that of CTZ against a wide range of gram-positive and gram-negative species. CTZ-R6 even gained activity against enterococci, which generally exhibit intrinsic cephalosporin resistance due to low-affinity penicillin-binding proteins and the production of β-lactamases (18, 19). In mice, CTZ-R6 demonstrated therapeutic efficacy against a vancomycin-resistant *Enterococcus faecium* strain, reducing the bacterial count in the liver, whereas CTZ did not (18). These findings could be attributed to a change in the biodistribution profile, since CTZ-R6 accumulated primarily in the liver and, to a lesser extent, in the spleen and kidney (18). In addition, the preliminary safety profile was encouraging. CTZ-R6 did not lyse human red blood cells up to a concentration of 320 µg·mL^−1^, the metabolic activity of human kidney or liver cells was not impaired at 512 µg·mL^−1^, and no compound-related adverse effects were noted in the biodistribution and *in vivo* efficacy studies.

In the current study, we explored the mode of action of CTZ-R6, differentiating between gram-positive and gram-negative bacteria and dissecting the contributions of the β-lactam and the polycationic peptide to the activity of the conjugate.

## RESULTS

### CTZ-R6 surpasses the activity of CTZ against multi-resistant gram-negative clinical isolates

We have recently shown that, compared to the parent β-lactam CTZ, CTZ-R6 exhibited increased activity against gram-positive and gram-negative bacteria, particularly against enterococci and *B. subtilis* (18). Here, we confirmed a 500-fold increased activity against *B. subtilis* and a 4-fold improvement against *Staphylococcus aureus*. In gram-negative bacteria, the factor of improvement was 4 on average against Enterobacteriaceae and 16–32 against non-fermenting bacteria (Table 1). These factors relate to the µg·mL^−1^ values used by convention for MIC determinations. Considering the higher molecular weight of the conjugate (1,783.12 g·mol^-1^) compared to CTZ (547.58 g·mol^-1^), the molar factor of improvement is even three times higher (Table S2). We investigated the efficacy of the conjugate against recent clinical multi-resistant isolates categorized as 3MRGN or 4MRGN according to the German classification system for gram-negative rod-shaped bacteria (20). Briefly, a bacterial strain is classified as 4MRGN if it is resistant against acylaminopenicillins, third or fourth generation cephalosporins, carbapenems, and quinolones or contains a carbapenemase and is classified as 3MRGN if it is still sensitive to one of the listed antibiotics and contains no carbapenemase. The conjugate sensitized some multi-resistant isolates to MIC values (in µg·mL^−1^) below the established clinical breakpoints that define susceptibility to CTZ (21).

**TABLE 1:** Antimicrobial activity of CTZ and CTZ-R6 against different gram-positive and negative strains

Bacterial taxonomy	Bacterial species	CTZ breakpoint (EUCAST)	MRGN^*a*^ classification	β-lactamase/carbapenemase	MIC (µg·mL^−1^)^*b*^		Factor of improvement
					CTZ	CTZ-R6	
**Gram-positive**							
Bacilli	*Bacillus subtilis* 168	S	–^*e*^	–^*f*^	2	0.004	500
Cocci	*Staphylococcus aureus* ATCC 29213	^*d*^	–	–	16	4	4
	*Staphylococcus aureus* USA300 JE2	^*d*^	MRSA	–	64	16	4
**Gram-negative**							
Enterobacteriaceae	*Escherichia coli* BW25113	S	–	–	0.25	0.0625	4
	*Escherichia coli* Tü20-025^*c*^	S	–	–	2	0.5	4
	*Escherichia coli* Tü21-027^*c*^	I	3MRGN	–	4	1	4
	*Escherichia coli* Tü21-031^*c*^	R	3MRGN	OXA244, ESBL	32	4	8
	*Escherichia coli* Tü21-032^*c*^	R	4MRGN	VIM, ESBL	>128	>128	0
	*Escherichia coli* Tü21-034^*c*^	R	4MRGN	OXA48, NDM	>128	>128	0
	*Klebsiella pneumoniae* Tü20-036^*c*^	S	–	–	0.125	0.0625	2
	*Klebsiella pneumoniae* Tü20-050^*c*^	R	4MRGN	KPC	>128	>128	0
	*Citrobacter freundii* Tü21-052^*c*^	S	–	–	0.25	0.125	2
	*Citrobacter freundii* Tü21-063^*c*^	R	3MRGN	ESBL	32	8	4
	*Citrobacter freundii* Tü21-067^*c*^	R	3MRGN	AmpC	64	0.5	128
	*Citrobacter freundii* Tü20-080^*c*^	R	4MRGN	VIM	>128	64	>2
Non-fermenters	*Pseudomonas aeruginosa* ATCC 27853	S	–	–	1	0.25	4
	*Pseudomonas aeruginosa* Tü20-021^*c*^	I	3MRGN	–	32	2	16
	*Pseudomonas aeruginosa* Tü20-011^*c*^	S	3MRGN	–	4	1	4
	*Pseudomonas aeruginosa* Tü20-012^*c*^	R	4MRGN	VIM	64	16	4
	*Acinetobacter baumannii* Tü21-037^*c*^	^*d*^	–	–	64	4	16
	*Acinetobacter baumannii* Tü21-035^*c*^	^*d*^	–	–	128	8	16
	*Acinetobacter pittii* Tü21-041^*c*^	^*d*^	–	–	4	0.125	32
	*Acinetobacter baumannii* Tü21-047^*c*^	^*d*^	4MRGN	–	>128	8	>16
	*Acinetobacter baumannii* Tü20-079^*d*^	^*d*^	4MRGN	OXA23	128	16	8

^*a*^ Multidrug-resistant gram-negative, rod-shaped bacteria that are resistant against three or four (i.e., 3MRGN or 4MRGN, respectively) of the following antibiotics: penicillins, cephalosporins, carbapenems, and fluoroquinolones (German classification to monitor the emerging antibiotic resistance crisis) (20).

^*b*^ MIC values (µg·mL^−1^) are presented as mode values (i.e., the concentration that appeared most often in at least three biological replicates).

^*c*^ Clinical isolate.

^*d*^ No EUCAST clinical breakpoint (21).

^*e*^ Not classified as MRGN.

^*f*^ Contains no β-lactamase/carbapenemase.

Despite its overall improved activity, CTZ-R6 was less active than CTZ against bacterial strains belonging to the family Morganellaceae (Table S2). This pattern was consistent across all tested species, including *Proteus* spp., *Providencia* spp., and *Morganella* spp.

In addition to its use as an individual agent, CTZ is important as a reserve antibiotic in a fixed combination with avibactam (11), the first diazabicyclooctane non-β-lactam β-lactamase inhibitor with a broad target spectrum (e.g., class A and C serine β-lactamases and carbapenemases, including KPC). AVI similarly improved the activity of CTZ-R6 and CTZ against Enterobacteriaceae (Table S3), providing the first indication that CTZ-R6 retains characteristics of CTZ with respect to its mode of action.

Of further mechanistical relevance was our observation that covalent linkage between CTZ and the hexa-arginine moiety is essential for potent activity. An equimolar mixture of R6C and CTZ as separate components was not more active than CTZ alone (18). The BB linker itself did not show any activity, and the separate hexa-arginine peptide R6C exhibited only weak, clinically irrelevant antibacterial activity (Table S4 [18]). Regarding the stereochemistry of the conjugate, we did not note a marked difference in antibacterial activity between the L-form (CTZ-L-R6) and the D-form (CTZ-D-R6) of the hexa-arginine moiety (Table S5). Due to the higher cost of synthesizing CTZ-D-R6, the corresponding L-form was used throughout the study.

A fourth point of mechanistic interest was that monovalent cations inhibited CTZ-R6 activity. We noted that in Mueller-Hinton II broth (MHB II), the standard medium for MIC determination and low in monovalent cations, CTZ-R6 was about 16 times more effective than in Luria-Bertani (LB) broth, which contains 1% NaCl. When the NaCl concentration in LB was lowered, antibacterial activity was restored to the MHB II level (Table S5). KCl (1%) showed the same effect as NaCl (Table S6). Cationic antimicrobial peptides are commonly antagonized by salts (22), providing a first indication that the hexa-arginine moiety also contributes to the antibacterial potency of the conjugate.

Along the same line, our data suggest that the activity of the conjugate is influenced by bacterial surface charge. In a series of isogenic *S. aureus* mutants varying in their contents of lipoteichoic acid (LTA), wall teichoic acid (WTA), and phosphatidylglycerol, as well as the degree of LTA and WTA D-alanylation, we observed up to a 500-fold improved antibacterial potency in mutants with a more negatively charged cell envelope than the wild type, e.g., *S. aureus* SA113 Δ*dltA* (23) and Δ*mprF* (24) (Table S7). Notably, neither a complete loss of WTA nor a strong reduction (by 87%) in LTA reduced the activity of the conjugate, indicating robustness of CTZ-R6 to lowering the negative charge at the cytoplasmic membrane and at peptidoglycan-linked glycopolymers. In addition, CTZ-R6 was unaffected by MprF mutations conferring daptomycin resistance (24).

### CTZ-R6 damages the integrity of the peptidoglycan sacculus and triggers slow lysis

**Fig 2:**
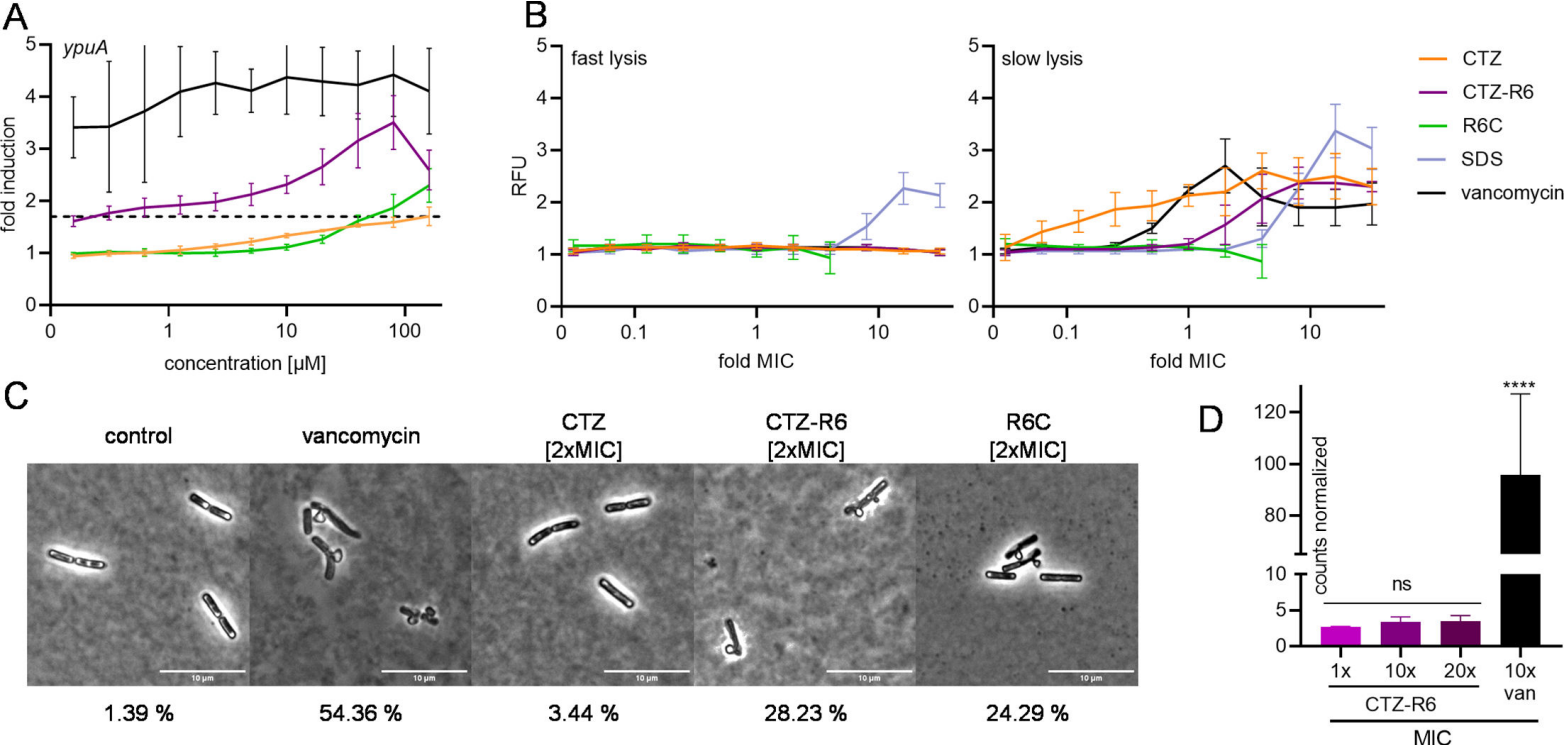
CTZ-R6 causes structural damage to the cell wall. (**A**) Luminescence emitted by the cell-envelope stress reporter *ypuA* in *B. subtilis*. Induction threshold was 1.7-fold above the untreated control (dashed line). Vancomycin was used as a positive control. Mean results for different cultures on different days (*n* = 3), with standard deviation (SD). All values are normalized to untreated cells. (**B**) BAU-reporter assay in *B. subtilis* to monitor lysis kinetics. Fast detergent-like lysis (measured after 5 min of compound exposure, left panel) and slow peptidoglycan synthesis inhibitor-like lysis (measured after 60 min, right panel) are observed. Vancomycin was used as a positive control (*n* = 3, ±SD; normalized to the untreated control). (**C**) Membrane “blebs” protruding through areas of weakened peptidoglycan in *B. subtilis* after 15 min of compound exposure. Vancomycin was used as a positive control, *n* = 3, scale bar, 10 µm. Representative images and fraction of cells showing blebs among >100 cells analyzed per condition. (**D**) Quantification of the ultimate cytoplasmic peptidoglycan precursor UDP-MurNAc-pentapeptide in *S. aureus* by LC/MS (*n* = 3, mean ± SD, normalized to the untreated control). Vancomycin was used as a positive control. ****, *P* < 0.0001 (one-way ANOVA).

To unravel which moiety conveys which characteristics to the hybrid, we compared the activities of the conjugate to those of its components when tested as separate entities, i.e., the β-lactam CTZ on the one hand, and the hexa-arginine R6C on the other hand. For an initial unbiased assessment of the type of stress imposed on treated cells, we used a bioreporter panel previously validated against a wide range of reference antibiotics (25). Several isogenic *B. subtilis* bioreporter strains express firefly luciferase from different promoters, which are selectively induced by antibiotics with distinct modes of action. Like β-lactams, CTZ-R6 induced the *ypuA* promoter (Fig. 2A), which signals general cell envelope stress (25). In contrast, the *liaI* promoter, which is more specifically triggered by inhibitors of membrane-associated stages of peptidoglycan synthesis (“lipid II cycle”), was not affected, nor was there any indication of DNA stress, RNA synthesis inhibition, or translation stalling (Fig. S1). In addition, R6C alone induced *ypuA* (Fig. 2A), indicating that the hexa-arginine molecule can also disturb the bacterial cell envelope, as reported for polycationic peptides (26). However, R6C showed this effect only at significantly higher molarities than the conjugate. When tested against BAU-102, a *B. subtilis* bioreporter strain yielding a strong β-galactosidase signal upon lysis (27), we observed delayed lysis for the conjugate (after 60 min of treatment) in agreement with the activity of a peptidoglycan synthesis inhibitor requiring cell growth to take effect, while rapid, detergent-like lysis could be excluded (Fig. 2B). In line with these observations, the peptidoglycan sacculus of *B. subtilis* was weakened by the conjugate, a defect visualized after only 15 min of exposure to CTZ-R6 by fixation with acetic acid/methanol (28). During this procedure, the cytoplasmic membrane is extruded through areas of weakened peptidoglycan, thereby forming blebs (Fig. 2C). The percentage of cells exhibiting blebs at this early stage was approximately 25% and similar for CTZ-R6 and R6C, while CTZ induced a similar phenotype only after a prolonged treatment of 60 min. Analysis of the soluble peptidoglycan precursor pool yielded a β-lactam-conform profile (Fig. 2D; Fig. S2). Unhindered production of the ultimate soluble peptidoglycan precursor UDP-*N*-acetyl muramic acid pentapeptide (UDP-MurNAc-PP) excluded interference with cytoplasmic reactions of peptidoglycan synthesis. At the same time, the lack of UDP-MurNAc-PP accumulation suggested a mode of action different from that of inhibitors targeting membrane-associated stages, such as vancomycin. Overall, our observations suggest inhibition of peptidoglycan synthesis with β-lactam-like features and accelerated peptidoglycan weakening enabled by the polycationic peptide.

### CTZ-R6 enters gram-negative bacteria by self-promoted uptake

**Fig 3:**
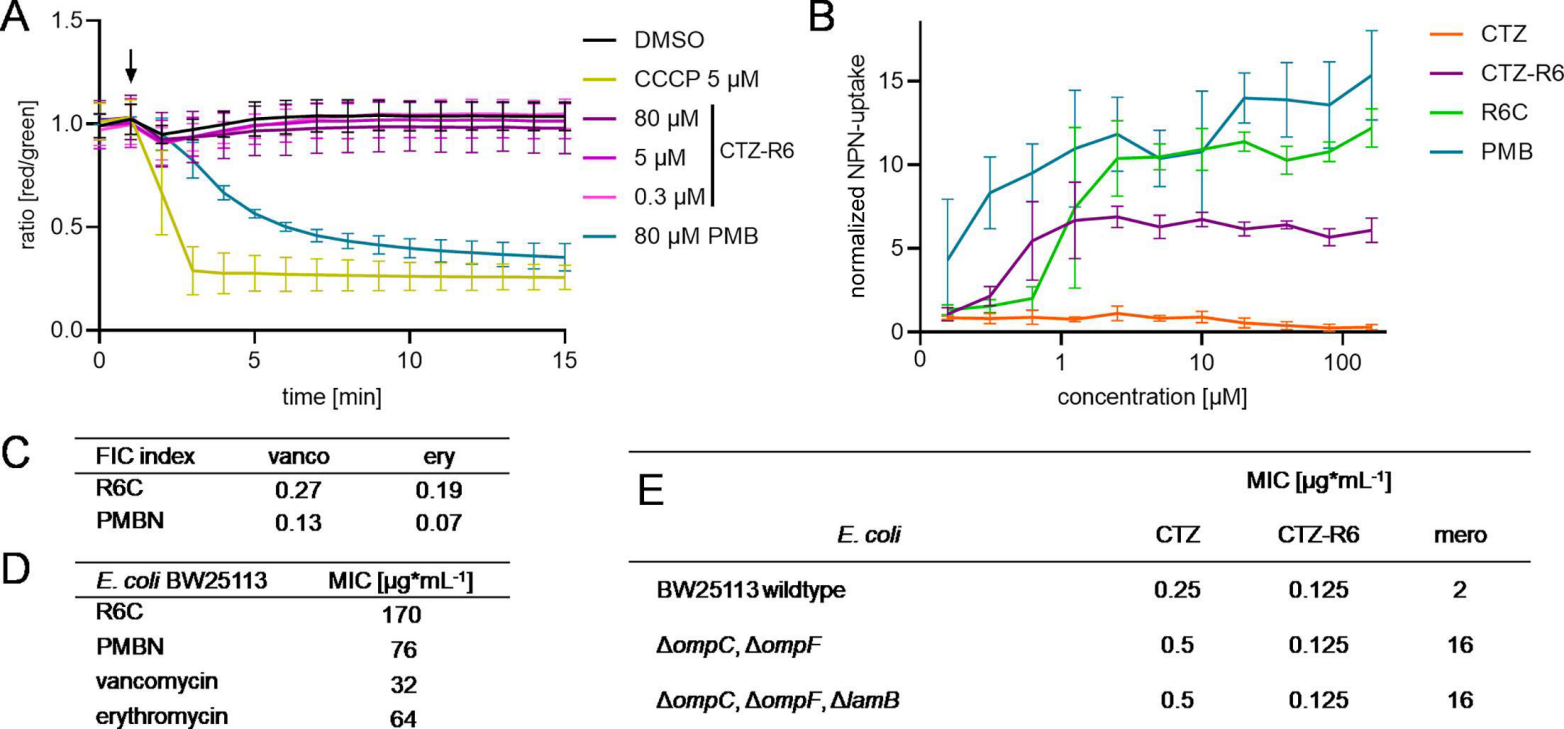
CTZ-R6 does not damage the cytoplasmic membrane but permeabilizes the outer membrane of gram-negative bacteria. (**A**) Potential difference at the cytoplasmic membrane in *B. subtilis* measured with 3,3′-diethyloxacarbocyanine iodide, which undergoes a red-to-green shift upon membrane potential dissipation (*n* = 3, ± SD). DMSO (1%) was used as a negative control, and carbonyl cyanide-m-chlorophenylhydrazone (CCCP) (5 µM) was used as a positive control. Arrow marks compound addition. PMB, polymyxin B. (**B**) Passage of the fluorophore 1-N-phenyl-naphthylamine (NPN) through the permeabilized outer membrane of *E. coli* (*n* = 3, normalized to the untreated control, ±SD). DMSO (1%) was used as a negative control, and PMB was used as a positive control. (**C**) Fractional inhibitory concentration (FIC) index calculated from checkerboard assays in *E. coli* BW25113. FIC < 0.5 indicates synergism. (**D**)  MICs as mode values (*n* = 3) for the compounds used in the checkerboard assay. In panel D, compounds were tested as separate agents; in panel **C**, they were combined. (**E**) MICs as mode values (*n* = 3) for *E. coli* BW25113 and isogenic, porin-deficient mutants. The positive control meropenem permeates through porins (e.g., OmpF and OmpC in *E. coli* [30]).

Next, we explored the impact of CTZ-R6 on the integrity and function of the bacterial inner and outer membranes. In *B. subtilis*, we measured the membrane potential, i.e., the electrochemical difference generated by ion gradients, across the cytoplasmic membrane. Even at CTZ-R6 concentrations orders of magnitude above the MIC, the membrane potential remained intact during the first 15 min of treatment (Fig. 3A; Fig. S3), excluding cytoplasmic membrane disruption and even small pores as the primary cause of death. In contrast, polymyxin B, used as a positive control in this assay, caused profound depolarization, in accordance with previous reports (29).

In contrast, the barrier function of the outer membrane of *Escherichia coli* was strongly impaired by CTZ-R6 (Fig. 3B), allowing entry of the fluorescent dye 1-N-phenyl-naphthylamine (NPN), which cannot pass an intact lipopolysaccharide (LPS) layer. This outer membrane-permeabilizing characteristic of the conjugate was caused by the R6C peptide, which also exhibited this feature when applied as a separate entity, while CTZ did not. The R6C peptide alone also permeabilized the outer membrane of *E. coli* to vancomycin and erythromycin (Fig. 3C), antibiotics too large to pass this barrier when intact (Fig. 3D). This activity of R6C resembles that of polymyxin B nonapeptide (PMBN), a PMB congener lacking the hydrophobic side chain of the antibiotic. PMBN is devoid of significant antibacterial activity but contains the potent LPS-permeabilizing capacity of PMB (31). These results indicate that the CTZ-R6 conjugate crosses the outer membrane of gram-negative cells via self-promoted uptake, a capacity also known for PMB (32). Accordingly, CTZ-R6 did not depend on porins, which are entry routes for most β-lactams (Fig. 3E). Importantly, the activity of CTZ-R6 was not affected by the plasmid-borne globally disseminated colistin (polymyxin E) resistant determinant MCR-1. In an isogenic set of *E. coli* strains, the expression of MCR-1, an enzyme that attaches phosphoethanolamine to lipid A, thereby reducing the net negative charge of LPS (33), raised the MIC of colistin by 10-fold but did not reduce susceptibility to CTZ-R6 (Table S8).

### CTZ-R6 and CTZ target PBPs of *B. subtilis* with different efficacy

**Fig 4:**
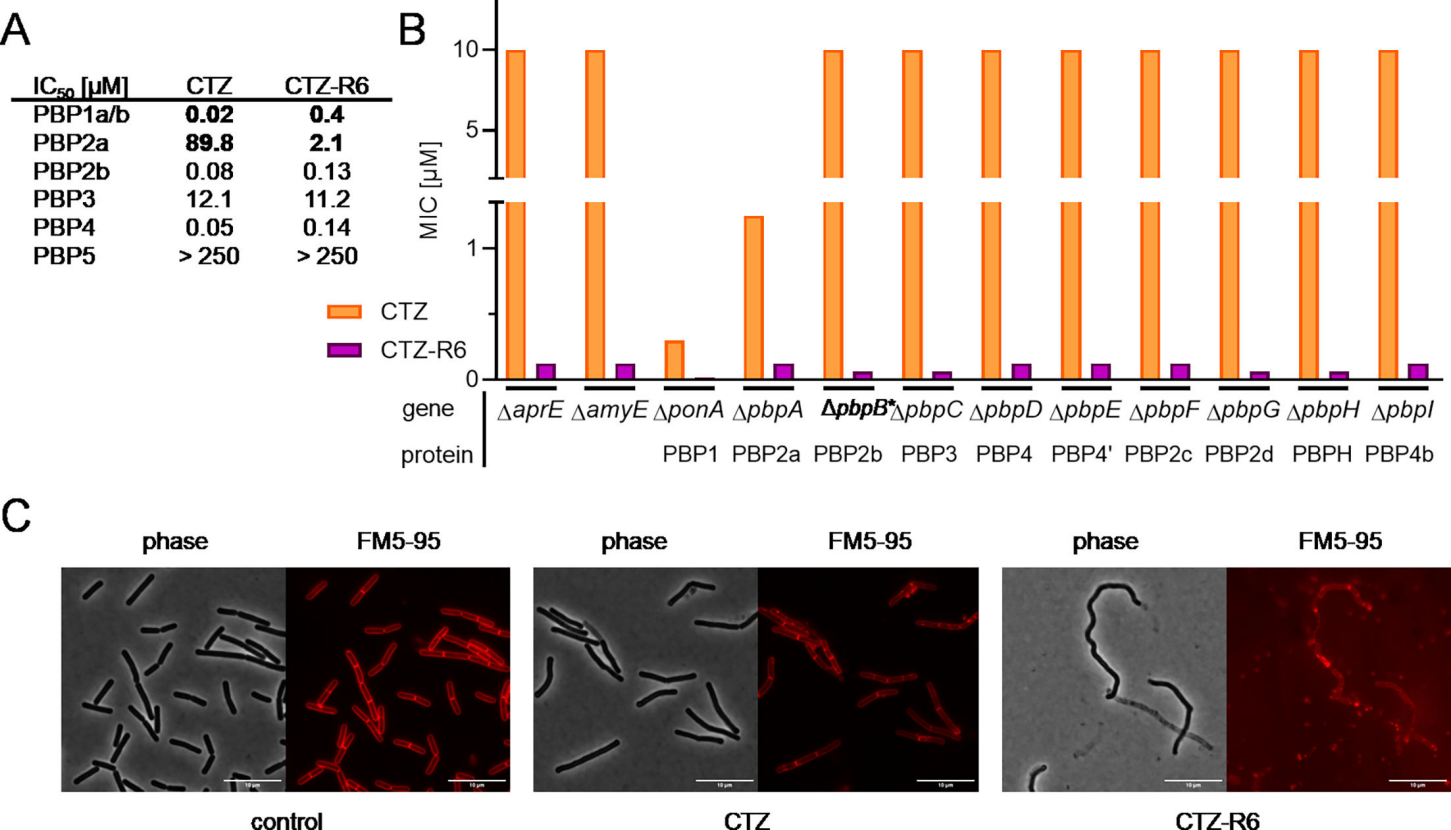
CTZ-R6 and CTZ target PBPs differently. (**A**) Binding profile to *B. subtilis* PBPs. IC50 values (calculated from the data published by Werner et al. [18]) represent the compound amounts required to competitively reduce binding of Bocillin FL by 50%. (**B**) MICs as mode values (*n* = 3) for isogenic PBP-deficient *B. subtilis* knockout mutants and a xylose-inducible knockdown mutant of essential PBP2b (*). As the *B. subtilis* full genome knockout library does not contain an unmutated wild type, strains Δ*amyE* and Δ*aprE* from the same strain library were used as wild-type-like reference. Both genes are commonly used for insertions in genetic engineering, and their loss is without relevance for *B. subtilis* under most conditions. MIC values (µM) are presented as mode values. (**C**)  Morphological aberrations induced by CTZ-R6, but not CTZ, in *B. subtilis* after 120 min at 1× MIC. Scale bar, 10 µm.

To better understand the exceptional increase in the potency of CTZ-R6 relative to CTZ in *B. subtilis*, we focused on penicillin-binding proteins, the targets of β-lactams. In our previous study, we observed differential binding of CTZ-R6 versus CTZ to the set of *B. subtilis* PBPs (18). In an assay measuring the competitive displacement of the fluorescent penicillin derivative Bocillin FL, CTZ competed most efficiently for binding to PBP1a/1b (the unprocessed/processed form of PBP1), PBP2b, and PBP4. The interaction profile of CTZ-R6 was overall similar to that of the parent β-lactam, with the notable differences that binding to PBP1a/b decreased by about 20-fold and binding to PBP2b increased by 45-fold (Fig. 4C [18]). Here, we determined MIC values for isogenic single-gene knockout mutants of all PBPs of *B. subtilis* but the essential PBP2b, for which we tested the effect of gene silencing by CRISPRi (Fig. 4B). Notably, the absence of PBP1 sensitized *B. subtilis* more than 30-fold to CTZ, while the potency of CTZ-R6 was not markedly affected by the loss of a single PBP. In addition, the phenotype of *B. subtilis* differed when exposed to the conjugate versus parent β-lactam (Fig. 4C). After 2 h of incubation with CTZ-R6 at 1× MIC, cells that were not yet lysed showed an elongated, crooked phenotype, in contrast to CTZ-treated cells. In addition, the fluorescent membrane dye FM 5-95 was irregularly dispersed in the membrane of conjugate-exposed cells, indicating greater disturbance of overall membrane topology than in CTZ-exposed cells.

## DISCUSSION

New concepts for extending the lifespan of clinically important antibiotics are essential. The hexa-arginine moiety R6C showed promise *in vitro* and in animal models of infection when conjugated via linkers to different antibiotic classes. R6C sensitized vancomycin-resistant enterococci to the glycopeptide (17) and daptomycin-resistant *S. aureus* to the lipopeptide (34). When attached to all four sub-classes of β-lactams, it increased their potency, particularly that of ceftazidime (18).

Here, we dissected the contribution of the individual moieties of the CTZ-R6 conjugate to the increased potency, showing that both the CTZ and R6C regions contribute to antibacterial activity. Despite lacking clinically relevant potency, R6C is essential for the activity of the conjugate against gram-negative bacteria. In the large conjugate, CTZ loses its capacity to cross the outer membrane through porins. The polycationic hexa-arginine moiety compensates this loss by enabling passage through the LPS layer and serving as a carrier for CTZ as a conjugated cargo. Our data show that outer membrane disruption by R6C is almost as efficient as by polymyxins, which serve as a reference for self-promoted uptake (35). Agents with multiple positive charges can bind to the negatively charged phosphates in the lipid A and core sugar region of LPS and displace the stabilizing divalent cations Mg^2+^ and Ca^2+^ (36). R6C not only efficiently mediates the translocation of the conjugate across the outer membrane but also that of other large antibiotics such as vancomycin and erythromycin, which can permeate as unconjugated entities.

Upon entering the periplasm, CTZ has access to its targets, the penicillin-binding proteins located at the cytoplasmic membrane. Covalent PBP inhibition prevents transpeptidation, consistent with the observed weakening of the peptidoglycan sacculus and cell lysis. At the cytoplasmic membrane, R6C shows its second strength. Cationic antimicrobial peptides are known to interact with negatively charged lipids in bacterial cytoplasmic membranes (26). The strong antagonizing effect of Na^+^ and K^+^ (15-fold reduction of activity in the presence of 1% monovalent ions) reflects this interaction. We conclude that the hexa-arginine moiety concentrates the conjugate at the cytoplasmic membrane, thereby positioning the β-lactam warhead of CTZ in close vicinity of its targets. In addition, the R6C moiety perturbs membrane topology, as evidenced by the irregular distribution of dye molecules, thereby affecting the composition and movement of large biosynthetic complexes within the membrane, likely including the peptidoglycan synthesis machinery. Those two effects of R6C on the cytoplasmic membrane affect bacteria broadly and may account for the four- to eightfold general increase in activity observed across the phylogenetic spectrum.

In some species, additional effects contribute to the conjugate’s potency. *B. subtilis* is 500-fold more sensitive to CTZ-R6 than to the parent CTZ, which could be explained by their differential PBP engagement. Conjugation to R6C changed the ability of the CTZ warhead to compete with the β-lactam Bocillin FL for binding to the PBPs in a *B. subtilis* membrane fraction (18). Among the high-molecular-weight PBPs, CTZ, as a separate entity, demonstrated a strong preference for PBP1 and PBP2b and a low affinity for PBP2a, whereas in the conjugate, the CTZ moiety showed a more balanced engagement of all three PBPs. In line with this, the absence of single PBPs affected the MICs of the conjugate less strongly than those of the free parent CTZ. The more balanced PBP-binding profile of CTZ-R6 could also be related to the increased membrane affinity conferred by the R6C moiety. Higher inhibitor concentrations and longer exposure times support covalent inactivation of a target with lower affinity.

Regarding known resistance mechanisms, two aspects appear relevant given the polycationic nature of CTZ-R6 and its interactions with negatively charged components of the bacterial cell envelope. Polymyxin resistance is mediated by enzymes that reduce the negative charge of LPS. Here, we show that the interaction of RC6 with the outer membrane of *E. coli* is not diminished by the globally disseminated, *mcr-1*-encoded LPS modification that confers colistin resistance. In addition, the reduction of the negative charge of the cytoplasmic membrane and in peptidoglycan-bound glycopolymers in *S. aureus* did not reduce susceptibility to the conjugate, whereas many cationic antimicrobial peptides are affected by these mechanisms (37). On the other hand, despite the broad target spectrum of CTZ-R6 and its relative robustness to the resistance determinants discussed above, Morganellaceae appear to be intrinsically resistant. In *Proteus* spp., intrinsic resistance to polymyxins is attributed to LPS decoration by diverse positive moieties (38). It might be that the diverse modifications realized in this species, such as concomitant attachment of 4-amino-4-deoxy-L-arabinose and phosphoethanolamine (39), inhibit the interaction with CTZ-R6. In addition, we cannot exclude a potential PBP configuration in Morganellaceae with lower affinity to the conjugate.

In summary, our mode-of-action analysis shows that CTZ-R6 is more than the sum of its parts. In our previous study, we reported that only covalently linking CTZ and R6C in the conjugate, but not mixing them as separate entities, increases potency (18). Here, we offer the mechanistic explanation. The conjugate retains the characteristics of the parent β-lactam CTZ and benefits from the R6C moiety in two ways. The hexa-arginine serves, on the one hand, as a vehicle that efficiently transports CTZ across the outer membrane, thereby rendering it independent of porin downregulation, a common resistance mechanism of β-lactams. On the other hand, the hexa-arginine serves as an anchor to concentrate the conjugate at the cytoplasmic membrane, enabling efficient PBP engagement and cell lysis via peptidoglycan rupture, likely enhanced by a general disturbance of membrane topology. Notably, the electrochemical gradient across the cytoplasmic membrane remained intact in our study, indicating preserved membrane barrier function, in accordance with the absence of detergent-like lysis in bacterial cells observed here and the lack of hemolysis in erythrocytes observed earlier (18).

Our study confirms the potential of hexa-arginine conjugation to increase potency and extend the lifespan of clinically established antibiotic classes acting at the bacterial cell envelope. Benefits are evident across gram-positive and gram-negative pathogen families, and anti-gram-negative treatment regimens are further expanded by the opportunity to combine the conjugate with high-molecular-weight antibiotic classes, such as glycopeptides or macrolides, which are otherwise inactive against gram-negative bacteria.

## MATERIALS AND METHODS

### Synthesis of CTZ-R6 and its components

The conjugate CTZ-R6 (1,783.12 g·mol^−1^) containing CTZ, R6C, and a BB (6-maleinimidohexanamide) linker was synthesized as recently described (18). When used as separate entities in bioassays, the individual components had the following molecular masses: CTZ (547.58 g·mol^−1^), the BB linker (196.25 g·mol^−1^), and R6C (1,057.30 g·mol^−1^).

### Antibacterial activity

MICs were determined by broth microdilution according to the guidelines of the Clinical and Laboratory Standards Institute (40). A twofold serial dilution of the compounds was prepared in 50 µL cation-adjusted Mueller-Hinton broth (BD, Difco) in 96-well round-bottom polystyrene microtiter plates (Sarstedt). Bacteria were added in 50 µL MHB II to yield a final inoculum of 5 × 10^5^ colony-forming units per mL. Plates were incubated for 20 h at 37°C. For combination experiments, 4 mg/L avibactam was added, as recommended by the EUCAST Clinical Breakpoint Tables (v. 13.0) for susceptibility testing (21). To assess media dependency, MICs in MHB II were compared with those in LB broth (1% tryptone and 0.5% yeast extract [pH 7.3]) with increasing salt concentrations (either NaCl from 0% to 1% or KCl at 1%). The lowest concentration preventing visible bacterial growth was considered the MIC. For growth curves, 96-well polystyrene microtiter plates (round bottom, Sarstedt) were prepared as described for the MIC assay and incubated for 24 h at 37°C in a microplate reader (TECAN Spark 10M). The optical density at 600 nm (OD_600_) was measured every 10 min.

The interaction between compounds was determined by the checkerboard assay. The fractional inhibitory concentration (FIC) index was calculated as (MIC_A combo_/_MICA alone_ + MIC_B combo_/MIC_B alone_), where A and B denote the interaction partners, MIC_combo_ refers to the MIC values for the combination, and MIC_alone_ refers to the MIC values of the compounds when tested individually. Synergism was defined as FIC < 0.5, additive effects as FIC 0.5–4, and antagonism as FIC > 4.

### Bioreporter assays

To detect the target area, a panel of isogenic bioreporter strains in sporulation-deficient *B. subtilis* IS34 was used, each expressing firefly luciferase from promoters validated to respond to different types of antibiotic stress (25). As described in reference 41, reporter strains were grown overnight at 37°C and 200 rpm, with 5 µg·mL^−1^ erythromycin as a resistance marker for the luciferase plasmid in either Belitsky minimal medium (42) (*bmrC* strain) or LB (Difco) (*yorB*, *helD*, *ypuA,* or *liaI* strains), diluted in fresh medium, and grown to an OD_600_ of 0.9, before adjusting cultures to an OD_600_ of 0.02. Serial twofold dilutions of test compounds (60 µL) were prepared in white 96-well flat-bottom polystyrene microtiter plates (Greiner) and inoculated with 60 µL of the bacterial suspension. Plates were incubated at 37°C for a duration reflecting the induction kinetics of the different reporter strains: 1 h for the *liaI* and *ypuA* strains, 1.5 h for the *helD* strain, 3.5 h for the *yorB* strain, and 4 h for the *bmrC* strain. Then, 60 μL of citrate buffer (0.1 M, pH 5) containing 2 mM luciferin (Serva) was added, and flash luminescence was recorded using a TECAN Spark 10M microplate reader. To distinguish between rapid and slow lysis, we used the bioreporter *B. subtilis* BAU-102, which expresses β-galactosidase from the *vanH* promoter, as described (27). Briefly, the strain was grown overnight in tryptone soy broth (TSB, Gibco) at 37°C and 190 rpm, with chloramphenicol (5 µg·mL^−1^) as a resistance marker for the plasmid. After dilution into fresh TSB and growth to an OD_600_ of 0.2, 10 mL of cells was harvested (4,098 × *g* at 4°C for 5 min) and resuspended in 10 mL of TSB. The suspension (90 µL per well) was added to black, flat-bottom 96-well plates (BRAND), containing 10 µL of compound in a twofold dilution series. DMSO (1%) in TSB served as the baseline. For the fast lysis setup, 10 µL of MUG dye (400 µg·mL^−1^, 4-methylumbelliferyl β-D-galactopyranoside in DMSO; Sigma-Aldrich) was added after 5 min at room temperature. For the slow lysis setup, cells were exposed to the compounds for 60 min at 37°C before MUG was added. Fluorescence was measured after 30 and 60 min (TECAN Spark 10M; excitation at 360 nm and emission at 455 nm).

### Fluorescence microscopy

*B. subtilis* 168 was grown overnight in LB at 37°C and 190 rpm and was used to inoculate fresh, prewarmed LB the day after. At an OD_600_ of 0.25, cells were treated with the different compounds or 1% DMSO as a negative control. At the indicated time points, cultures were mixed with the dyes and imaged immediately after spotting 0.5 µL onto 1% agarose-coated microscopy slides (43). Membranes were stained with FM5-95 [1 µg·mL^−1^ N-(3-trimethylammoniumpropyl)−4-(6-(4(diethylamino) phenyl) hexatrienyl) pyridinium dibromide, Thermo Fisher]. Images were acquired using a Nikon Eclipse Ti-E microscope and an Orca Flash 4.0 LT camera (Hamamatsu) with NIS Elements Advanced Research software (Nikon). For analysis and processing, NIS Elements and Fiji ImageJ were used.

### Peptidoglycan integrity

*B. subtilis* 168, grown overnight in LB at 37°C and 190 rpm, was used to inoculate fresh, prewarmed LB. At an OD_600_ of 0.5, cultures (100 µL) were treated with the compounds, vancomycin (4 µg·mL^−1^, positive control) or 1% DMSO (negative control). After 15 and 60 min at 37°C, 25 µL aliquots were added to a tube containing 100 µL of 1:3 (vol:vol) acetic acid and methanol (28). Images were acquired using a Nikon Eclipse Ti-E microscope and an Orca Flash 4.0 LT camera (Hamamatsu) with NIS Elements Advanced Research software (Nikon). For further analysis and processing, NIS Elements and Fiji ImageJ were used.

### Membrane potential

*B. subtilis* 168 was grown overnight in LB at 37°C and 190 rpm and was used to inoculate fresh, prewarmed LB. At an OD_600_ of 0.7, cells were harvested (4,000 × *g* for 5 min at 4°C) and resuspended to an OD_600_ of 0.5 in phosphate-buffered saline with 0.1% glucose. Cells were incubated for 15 min in the dark with 30 µM 3,3′-diethyloxacarbocyanine iodide (Molecular Probes, Fisher Scientific). Dye-loaded cells were transferred to a black flat-bottom 96-well polystyrene microtiter plate (BRAND). Fluorescence was measured using a TECAN Spark 10M, with an excitation wavelength of 485 nm and two emission wavelengths: 530 nm (green) and 630 nm (red) (44). First, a baseline measurement was taken for 2 min. Then, a twofold dilution series of compounds was added. The protonophore carbonyl cyanide-m-chlorophenylhydrazone (5 µM, CALBIOCHEM) served as a positive control, and 1% DMSO as a negative control.

### UDP-MurNAc-pentapeptide level

*S. aureus* ATCC 29213 was incubated overnight in LB at 37°C and 190 rpm and used to inoculate fresh, prewarmed LB. At an OD_600_ of 0.5, cells were treated with 130 µg·mL^−1^ chloramphenicol for 15 min. Aliquots of 5 mL were treated with the compounds at the indicated concentrations for 30 min or left untreated as a control. Samples were cooled on ice, pelleted, resuspended in 500 mL of demineralized water, and disrupted by 0.1 mm glass beads in a Precellys Evolution homogenizer (3 × 20 s, 6,500 rpm, with 30-s interval). After centrifugation (16,873 × *g* at 4°C for 20 min), the supernatants (400 µL) were transferred to a fresh tube containing 1.5 mL of HPLC-grade acetone. As described in reference 45, the samples were again centrifuged for 15 min, and the supernatant was transferred to a fresh tube and dried in a vacuum centrifuge. Samples were resuspended in 50 µL of demineralized water, and 5 µL of the suspension was analyzed by the LC/MSD Ultra Trap System XCT 6330 (Agilent, Waldbronn) using a Gemini-110 C18 column (150 × 2 mm ID, Phenomenex, Aschaffenburg) and a UltiMate 3000 RS (Dionex) coupled to a MicrO-TOF II mass spectrometer (Bruker) operated in negative ion mode.

### NPN uptake

The outer membrane-impermeable dye NPN has a low fluorescence quantum yield in aqueous solution but emits bright fluorescence when it interacts with phospholipids of the cytoplasmic membrane (46). As it cannot pass an intact lipopolysaccharide layer, it can be used to detect leakiness of this barrier. *E. coli* BW25113 was grown overnight in 0.5% polypeptone (PP) at 37°C and 190 rpm, inoculated in fresh PP, and grown to an OD_600_ of 0.7. Twelve milliliters were harvested (3,000 × *g* for 5 min at 4°C), and the pellet was washed in 5 mM HEPES (2-[4-(2-hydroxyethyl)piperazine-1-yl]ethane-1-sulfonic acid) buffer (pH 7.5, ROTH) and resuspended in the same volume. Then, the cells were centrifuged (3,000 × *g* for 5 min at 4°C) and resuspended in 5 mM HEPES to an OD_600_ of 0.5. NPN (40 µM in 5 mM HEPES) was added to the cells and the culture was dispensed (50 µL per well) to a black flat-bottom 96-well microtiter plate (BRAND). A volume of 50 µL of antibiotic solution in 5 mM HEPES was added in a twofold dilution series. The plate was incubated for 5 min. Fluorescence was measured in a TECAN Spark 10M (excitation, 350 nm; emission, 420 nm). The intrinsic low fluorescence of NPN (NPN in buffer only) was subtracted from all samples to account for the background signal. To determine the permeation of NPN, the compound-treated samples were divided by the untreated samples (no antibiotic added). As positive controls, polymyxin B and polymyxin B nonapeptide, both known to permeabilize outer membranes, were used (46).

## Data Availability

The data sets used and analyzed during the current study are available from the corresponding author upon reasonable request.

## SUPPLEMENTAL MATERIAL

Supplemental Material spectrum.00693-26-s0001.pdf Tables S1 to S8; Fig. S1 to S3.

ASM does not own the copyrights to Supplemental Material that may be linked to, or accessed through, an article. The authors have granted ASM a non-exclusive, world-wide license to publish the Supplemental Material files. Please contact the corresponding author directly for reuse.
