# Supplementary material for: Synergistic mode of action of a ceftazidime-hexa-arginine conjugate with resistance-overcoming properties
Source: Microbiol Spectr. 2026 Jul 13;14(8):e00693-26. doi: 10.1128/spectrum.00693-26 (PMC13436345; doi:10.1128/spectrum.00693-26)
Supplement: Supplementary file 1 [file spectrum.00693-26-s0001.pdf]

Supplementary Material

Synergistic mode of action of a ceftazidime-hexa-arginine-conjugate with resistance-overcoming properties

Hannah Isabell Müller[1], Julia Werner[2], Julia Guther[3], Jan Straetener[1], Manuel Ben Böhmann[4], Philipp Uhl[2, 4], and Heike Brötz-Oesterhelt[1,5,6]*

1) Microbial Bioactive Compounds, Interfaculty Institute of Microbiology and Infection Medicine, University of Tübingen, Germany

2) Department of Nuclear Medicine, University Hospital Heidelberg, Germany

3) Institute of Medical Microbiology and Hygiene, University Hospital Tübingen, Germany

4) Department of Pharmaceutical Technology, Institute of Pharmacy and Molecular Biotechnology, University of Heidelberg, Germany

5) Cluster of Excellence Controlling Microbes to Fight Infection, Tübingen, Germany

6) German Center for Infection Research, Partner Site Tübingen, Germany

* Corresponding author: Heike Brötz-Oesterhelt, Microbial Bioactive Compounds, Interfaculty Institute of Microbiology and Infection Medicine, University of Tübingen, Auf der Morgenstelle 28, 72076 Tübingen, Germany
Phone: +49 7071 2974706, Fax: +49 7071 295979
heike.broetz-oesterhelt@uni-tuebingen.de

Table of contents

Table S1 Bacterial strains used in this study. 2

Table S2 Antimicrobial activity of CTZ and CTZ-R6 in µM 4

Table S3 Antimicrobial activity of CTZ and CTZ-R6 combined with avibactam 5

Table S4 Antimicrobial activity of RC6 and the BB linker 5

Table S5 Antimicrobial activity of CTZ-L-R6 compared to CTZ-D-R6. 5

Table S6 Antimicrobial activity of CTZ and R6C in LB containing different concentrations of monovalent cations 6

Table S7 Antimicrobial activity of CTZ compared to CTZ-R6 against isogenic *S. aureus* mutants with varying surface charges.. 6

Table S8 Antimicrobial activity of CTZ compared to CTZ-R6, PMB and colistin against isogenic bacterial strains with and without colistin resistance 7

Figure S1 Bioreporter profiling 7

Figure S2 UDP-MurNAc-pp quantification 8

Figure S3 Membrane potential assay 8

Supplementary References 9

Table S1. Bacterial strains used in this study.

Strain	Information	Source
Bacillus subtilis 168	*trpC2*	[1]
Bacillus subtilis BAU-102	*trpC2*, *amyE*::(cat, P*vanH-lacZ*), *vanRS*	[2]
Bacillus subtilis 1S34 P_{ypuA}-luc	*B. subtilis ypuA* promoter-sequence upstream of the firefly luciferase gene in plasmid pHT304; *ypuA* induction signals cell envelope stress; *trpC2*, Spo-, pS107	[3, 4]
Bacillus subtilis 1S34 P_{bmrC}-luc	*B. subtilis bmrC* (formerly *yheI*) promoter-sequence upstream of the firefly luciferase gene in plasmid pHT304; *bmrC* induction signals translation stalling; *trpC2*, Spo-, pS72	[3, 4]
Bacillus subtilis 1S34 P_{yorB}-luc	*B. subtilis yorB* promoter-sequence upstream of the firefly luciferase gene in plasmid pHT304; *yorB* induction signals DNA stress; *trpC2*, Spo-, pS77	[3, 4]
Bacillus subtilis 1S34 P_{liaI}-luc	*B. subtilis liaI* promoter-sequence upstream of the firefly luciferase gene in plasmid pHT304; promoter assay; *liaI* induction signals lipid-II cycle stress in peptidoglycan synthesis; *trpC2*, Spo-, pS130	[3, 4]
Bacillus subtilis 1S34 P_{helD}-luc	*B. subtilis helD* (formerly *yvgS*) promoter-sequence upstream of the firefly luciferase gene in plasmid pHT304; *helD* induction signals RNA synthesis stress; *trpC2*, Spo-, pS63	[3, 4]
Bacillus subtilis 168 PBP knockout mutants	Whole genome single gene deletion library (non-essential genes replaced by a kanamycin resistance cassette)	[5]
Bacillus subtilis 168 PBP knockdown mutants	PBP knockdown mutant librarys	[6]
S. aureus ATCC29213	Wildtype, CLSI reference strain	American Type Culture Collection
S. aureus USA300JE2	MRSA, plasmid-cured USA300	[7]
S. aureus SA113	ATCC35556	American Type Culture Collection
S. aureus SA113 Δ*dltA*	Deletion of D-alanine-poly(phosphoribitol)ligase subunit 1	[8]
S. aureus SA113 *pRBdlt1*	Plasmid-encoded additional *dlt* operon	[8]
S. aureus SA113 Δ*tagO*	Deletion of N-acetylglucosamin-1-phosphate-transferase	[9]
S. aureus SA113 Δ*ypfP*	Deletion of diglucosyldiacylglycerol synthase involved in lipoteichoic acid (LTA) synthesis	[10]
S. aureus SA113 Δ*mprF*	Deletion of LysPG synthase/flippase *MprF*	[11]
S. aureus SA113 *mprF* T345A	Gain of function mutation in LysPG synthase/flippase *MprF* that confers specific resistance to daptomycin-type antibiotics	[11]
S. aureus SA113 *mprF* V351E	Gain of function mutation in LysPG synthase/flippase *MprF* that confers specific resistance to daptomycin-type antibiotics	[11]
E. coli BW25113	Parent strain of the Keio collection<br>F-, Δ(*araD-araB*)567, Δ*lacZ*4787(::*rrnB*-3), λ-, *rph*-1, Δ(*rhaDrhaB*)568, *hsdR*514	[12]
E. coli BW25113 *ΔompC, ΔompF, ΔlamB*	Deletion of multiple porins	[13]
E. coli J53	Parent strain	[14, 15]
E. coli J53 pTOPO	Empty vector control	[14, 15]
E. coli J53 pTOPO *mcr-1 prom*	Plasmid containing *mcr-1*, phosphoethanolamine (pEtN) transferase (plasmid-encoded colistin resistance)	[14, 15]
P. aeruginosa ATCC27853	CLSI reference strain, originally isolated from blood culture (DSMZ-1117)	[16, 17]
E. coli Tü20-025 to Tü21-032	Clinical isolates from urine	Institute for Medical Microbiology and Hygiene, Tübingen
E. coli Tü21-034	Clinical isolate from blood culture	
K. pneumoniae Tü20-036, Tü20-050	Clinical isolates from urine	
C. freundii Tü20-080, Tü21-052, Tü21-063, Tü21-067	Clinical isolates from urine	
P. aeruginosa Tü20-011, Tü20-012, Tü20-021	Clinical isolates from urine	
A. baumannii Tü20-079, Tü21-035, Tü21-037, Tü21-047	Clinical isolates from urine	
A. pittii Tü21-041	Clinical isolates from urine	

P. mirabilis Tü20-066, Tü20-071, Tü20-073, Tü20-067	Clinical isolates from urine	
P. rettgeri VA13581, VB13369	Clinical isolates from wound smear	
P. rettgeri VB13628	Clinical isolate from bronchoalveolar lavage	
P. stuartii MRGN11081, MRGN12382	Clinical isolates from wound smear	
M. morganii VA13558, MRGN8836	Clinical isolates from wound smear	
M. morganii VA13721, VB13534, VB13623, MRGN8817	Clinical isolates from material of the respiratory system (sputum, tracheal secret, throat swab)	
M. morganii MRGN8901	Clinical isolates from anal swab	

Table S2. Antimicrobial activity of CTZ and CTZ-R6 against different Gram-positive and -negative strains depicted as compound molarities.

Bacterial taxonomy		Bacterial species	CTZ-breakpoint EUCAST	MRGN[1] classification	ß-lactamase / carbapenemase	MIC [µM][2]		Factor of improvement
						CTZ	CTZ-R6	
Gram-positive	Bacilli	*Bacillus subtilis* 168	S	-	-	3.7	0.002	1850
	Cocci	*Staphylococcus aureus* ATCC29213	[4]	-	-	40	2.5	16
		Staphylococcus aureus USA300JE2	[4]	MRSA	-	160	10	16
Gram-negative	Enterobacteriaceae	*Escherichia coli* BW25113	S	-	-	0.5	0.04	12.5
		Escherichia coli Tü20-025[3]	S	-	-	3.7	0.3	12
		Escherichia coli Tü21-027[3]	I	3MRGN	-	7.3	0.6	12
		Escherichia coli Tü21-031[3]	R	4MRGN	OXA244, ESBL	58.4	2.2	26.5
		Escherichia coli Tü21-032[3]	R	4MRGN	VIM, ESBL	> 233.8	> 71.8	0
		Escherichia coli Tü21-034[3]	R	4MRGN	OXA48, NDM	> 233.8	> 71.8	0
		Klebsiella pneumoniae Tü20-036[3]	S	-	-	0.2	0.04	5
		Klebsiella pneumoniae Tü20-050[3]	R	4MRGN	KPC	> 233.8	> 71.8	0
		Citrobacter freundii Tü21-052[3]	S	-	-	0.5	0.07	7
		Citrobacter freundii Tü21-063[3]	R	3MRGN	ESBL	58.4	4.5	13
		Citrobacter freundii Tü21-067[3]	R	3MRGN	AmpC	116.9	0.3	390
		Citrobacter freundii Tü20-080[3]	R	4MRGN	VIM	> 233.8	35.9	> 6.5
	Non-fermenters	*Pseudomonas aeruginosa* ATCC27853	S	-	-	1.8	0.1	18
		Pseudomonas aeruginosa Tü20-021[3]	I	3MRGN	-	7.3	0.6	12
		Pseudomonas aeruginosa Tü20-011[3]	S	3MRGN	-	58.4	1.1	53
		Pseudomonas aeruginosa Tü20-012[3]	R	4MRGN	VIM	116.9	9	13
		Acinetobacter baumanii Tü21-037[3]	[4]	-	-	7.3	0.07	104
		Acinetobacter baumanii Tü21-035[3]	[4]	-	-	116.9	2.2	53
		Acinetobacter pittii Tü21-041[3]	[4]	-	-	233.8	4.5	52
		Acinetobacter baumanii Tü21-047[3]	[4]	4MRGN	-	> 233.8	4.5	> 52
		Acinetobacter baumanii Tü20-079[3]	[4]	4MRGN	OXA23	233.8	9	26
	Morganellaceae	*Proteus mirabilis* Tü20-066[3]	S	-	-	0.2	35.9	0
		Proteus mirabilis Tü20-071[3]	I	3MRGN	-	7.3	> 71.8	0
		Proteus mirabilis Tü20-073[3]	R	3MRGN	ESBL	> 233.8	> 71.8	0
		Proteus mirabilis Tü20-076[3]	R	4MRGN	NDM	> 233.8	> 71.8	0
		Providencia rettgeri VB13369[3]	S	-	-	0.06	1.1	0
		Providencia rettgeri VB13628[3]	S	-	-	0.06	1.1	0
		Providencia rettgeri VA13581[3]	S	-	-	0.2	4.5	0
		Providencia stuartii MRGN11081[3]	R	3MRGN	ESBL	29.2	35.9	0
		Providencia stuartii MRGN12382[3]	R	4MRGN	NDM	> 233.8	> 71.8	0
		Morganella morganii VA13558[3]	S	-	-	0.2	18	0
		Morganella morganii VA13721[3]	S	-	-	0.9	4.5	0
		Morganella morganii VB13534[3]	I	-	-	1.8	4.5	0
		Morganella morganii VB13623[3]	I	-	-	0.06	18	0
		Morganella morganii MRGN8817[3]	R	-	AmpC	14.6	35.9	0
		Morganella morganii MRGN8836[3]	R	3MRGN	AmpC	58.4	18	0
		Morganella morganii MRGN8901[3]	R	3MRGN	AmpC	58.4	35.9	0

[1] Multidrug-resistant Gram-negative rod-shaped bacteria that are resistant against three or four (i.e., 3MRGN or 4MRGN, respectively) of the following antibiotics: penicillins, cephalosporins, carbapenems, fluorochinolones (German classification to monitor the emerging antibiotic resistance crisis) [18]
[2] MIC values [µM] are presented as mode values (i.e., the concentration that appeared most often in at least three biological replicates)
[3] clinical isolate
[4] no EUCAST clinical breakpoint [19]

Table S3. Antimicrobial activity of CTZ and CTZ-R6 in combination with avibactam (Avi) against different Gram-negative strains.

Bacterial species	ß-lactamase / carbapenemase	MIC [µg*mL^{-1}][1]			
		CTZ	CTZ + Avi	CTZ-R6	CTZ-R6 + Avi
E. coli BW25113	-	0.25	0.25	0.0625	0.0625
E. coli Tü21-027[2]	-	4	0.125	1	0.03
E. coli Tü21-031[2]	OXA244[3], ESBL[3]	32	0.25	4	0.015
E. coli Tü21-032[2]	VIM, ESBL[3]	> 128	> 128	> 128	32
E. coli Tü21-034[2]	OXA48[3], NDM	> 128	> 128	> 128	> 128
K. pneumoniae Tü20-036[2]	-	0.125	0.0625	0.0625	0.0625
K. pneumoniae Tü20-050[2]	KPC[3]	> 128	4	> 128	0.5

[1] MIC values [µg*mL^{-1}] are presented as mode values (i.e., the concentration that appeared most often in at least three biological replicates).
[2] clinical isolate
[3] approved for treatment with avibactam (formerly NXL104) [20, 21]

Table S4. Antimicrobial activity of R6C and the BB-linker against *B. subtilis* 168 and *E. coli* BW25113.

Cell wall composition	Bacterial species	MIC [µM][1]			
		CTZ	CTZ-R6	R6C	BB-linker
Gram-positive	*B. subtilis* 168	3.7	0.002	40	> 320
Gram-negative	*E. coli* BW25113	0.5	0.04	160	> 320

[1] MIC values [µM] are presented as mode values (i.e., the concentration that appeared most often in at least three biological replicates).

Table S5. Antimicrobial activity of CTZ-L-R6 compared to CTZ-D-R6 against representative bacterial strains used in the study.

Bacterial strain	MIC [µg*mL^{-1}][1]	
	CTZ-L-R6	CTZ-D-R6
B. subtilis 168	0.008	0.008
S. aureus ATCC 29213	2	1
S. aureus USA300 JE2	4	4
E. coli BW 25113	0.0625	0.0625
K. pneumoniae Tü20-036	0.125	0.125
C. freundii Tü21-052	0.25	0.25
P. aeruginosa ATCC 27853	1	0.5
A. baumannii Tü21-037	8	8

[1] MIC values [µg*mL^{-1}] are presented as mode values (i.e., the concentration that appeared most often in at least three biological replicates).

Table S6. Antimicrobial activity of CTZ and CTZ-R6 in LB with rising concentrations of NaCl and KCl, respectively, in comparison to MHB II. The standard recipe for LB contains 1% NaCl, whereas MHB II contains no added salts. Factor of antagonisation calculated for the difference between LB without salt and with 1% salt. Interestingly, and in contrast to CTZ-R6, the antibacterial activity of CTZ improved in the presence of Na^+ and K^+.

Bacterial species	Antibiotic	MIC [µM][1]						Factor of antagonisation
		MHB II	LB					
			NaCl				KCl	
			0%	0.25%	0.5%	1%	1%	
B. subtilis 168	CTZ	5	20	5	5	5	5	0.25
	CTZ-R6	0.008	0.008	0.016	0.0625	0.125	0.125	15.63

[1] MIC values [µM] are presented as mode values (i.e., the concentration that appeared most often in at least three biological replicates).

Table S7. Antimicrobial activity of CTZ compared to CTZ-R6 against isogenic *S. aureus* mutants with varying surface charge.

Strain name		Strain characteristics	Effects	Surface charge	MIC [µg*mL^{-1}][1]	
					CTZ	CTZ-R6
S. aureus SA113	Parent strain	ATCC wildtype	Comparator		16	2
	**Δ*dltA***	Deletion of D-alanine-poly(phosphoribitol)ligase subunit 1	Lack of D-alanylation of wall teichoic acids (WTA) and lipoteichoic acid (LTA)	More negative	8	**<0.004**
	pRBdlt1	Plasmid coded additional *dlt* operon	Increase of 5-18% D-alanine in LTA and WTA	Less negative	8	1
	Δ*tagO*	Deletion N-acetylglucosamine-1-phosphate-transferase	Lack of WTA	Less negative	16	4
	Δ*ypfP*	Deletion of diglucosyl diacylglycerol synthase	87% reduction of LTA content	Less negative	8	2
	**Δ*mprF***	Deletion of LysPG synthase/flippase MprF	More phosphatidyl glycerol (PG) and less lysyl-PG	More negative	8	**0.5**
	mprF T345A	Gain of function mutation in LysPG synthase/flippase MprF	Specific resistance to daptomycin-type antibiotics	No overall change	16	2
	mprF V351E	Gain of function mutation in LysPG synthase/flippase MprF	Specific resistance to daptomycin-type antibiotics	No overall change	8	1

[1] MIC values [µg*mL^{-1}] are presented as mode values (i.e., the concentration that appeared most often in at least three biological replicates).

Table S8. Antimicrobial activity of CTZ-R6 compared to CTZ, polymyxin B (PMB) and colistin against isogenic bacterial strains with and without colistin resistance.

	Strain name	Strain characteristics	Effects	Surface charge	MIC [µg*mL^{-1}][1]			
					PMB	colistin	CTZ	CTZ-R6
E. coli J53	Parent strain	*E. coli* K12 derivative	Comparator		0.125-0.25	≤0.06-0.125	0.25	0.03
	pTOPO	Empty vector control	Like comparator		≤0.06-0.125	≤0.06	0.25	0.06
	pTOPO *mcr-1*_prom	Plasmid containing *mcr-1*	Colistin resistance	Less negative	0.25-1	1	0.125	0.03

[1] MIC values [µg*mL^{-1}] are presented as mode values (i.e., the concentration that appeared most often in at least three biological replicates).

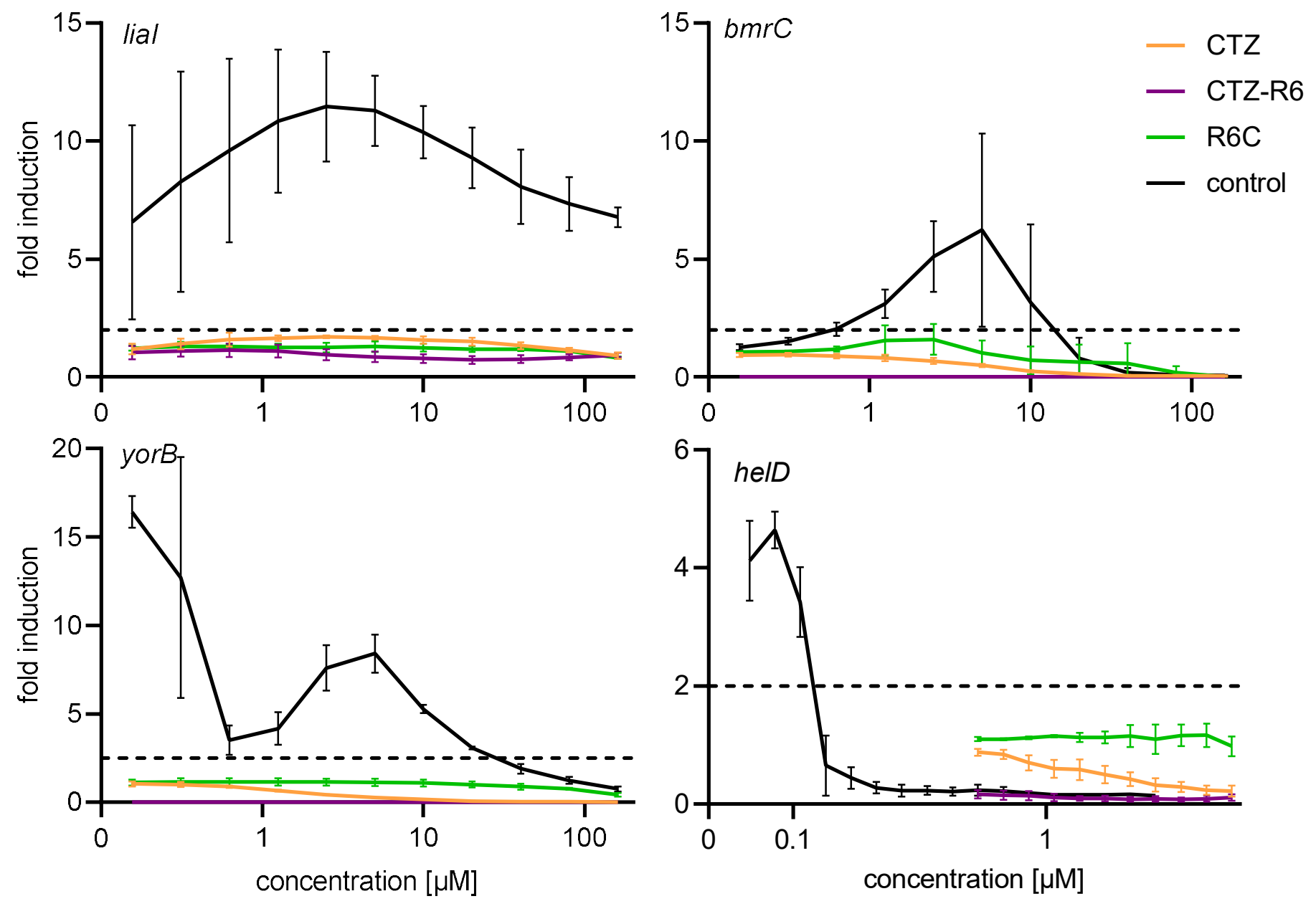

Figure S1. Bioreporter profiling of CTZ, CTZ-R6 and R6C to detect antibiotic-induced stress in *B. subtilis* (n=3, ± SD). None of the compounds induced *helD* signalling RNA synthesis inhibition, *yorB* signalling DNA stress, *bmrC* signalling translation stalling or *liaI* signalling lipid-II-cycle interference in peptidoglycan synthesis. The untreated control was set to 1. The dashed line shows the induction threshold as fold induction above the untreated control (2-fold for *liaI*, *bmrC* and *helD*, 2.5-fold for *yorB*). Positive controls were vancomycin (*liaI*), chloramphenicol (*bmrC*), ciprofloxacin (*yorB*) and rifampicin (*helD*). For *ypuA* signalling cell wall stress, which was induced, see Fig. 2B.

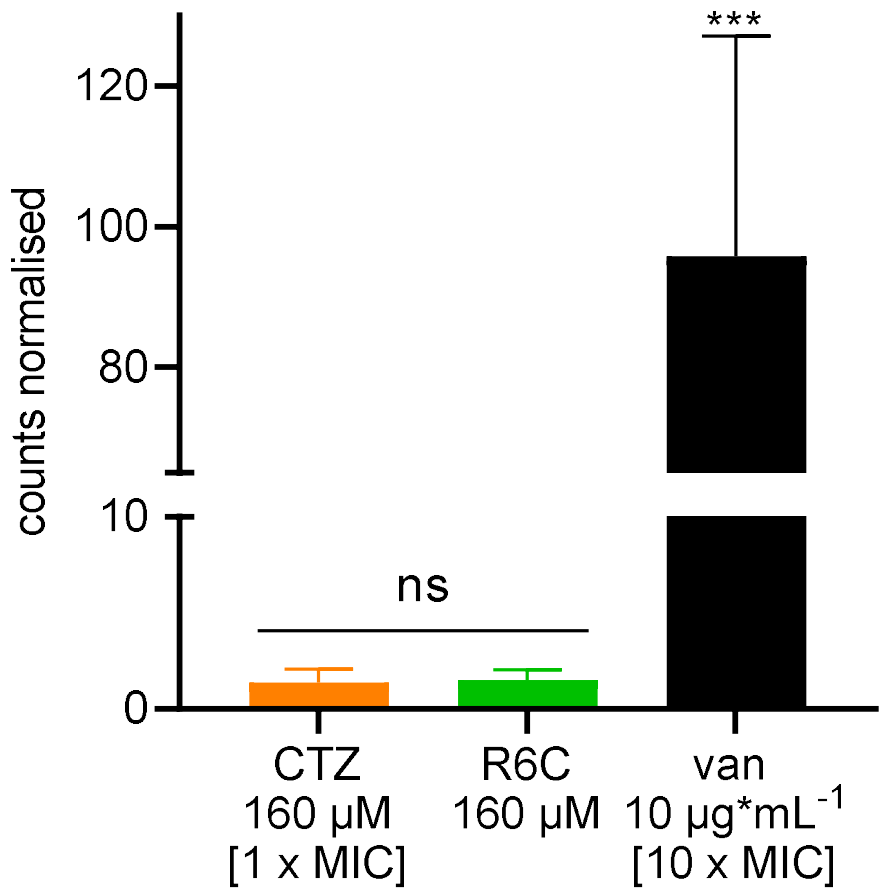

Figure S2. CTZ and R6C do not lead to accumulation of the peptidoglycan precursor UDP-MurNac-PP in *S. aureus* (n=3, ± SD). Vancomycin as positive control. ***, p = 0.0002 (one-way ANOVA).

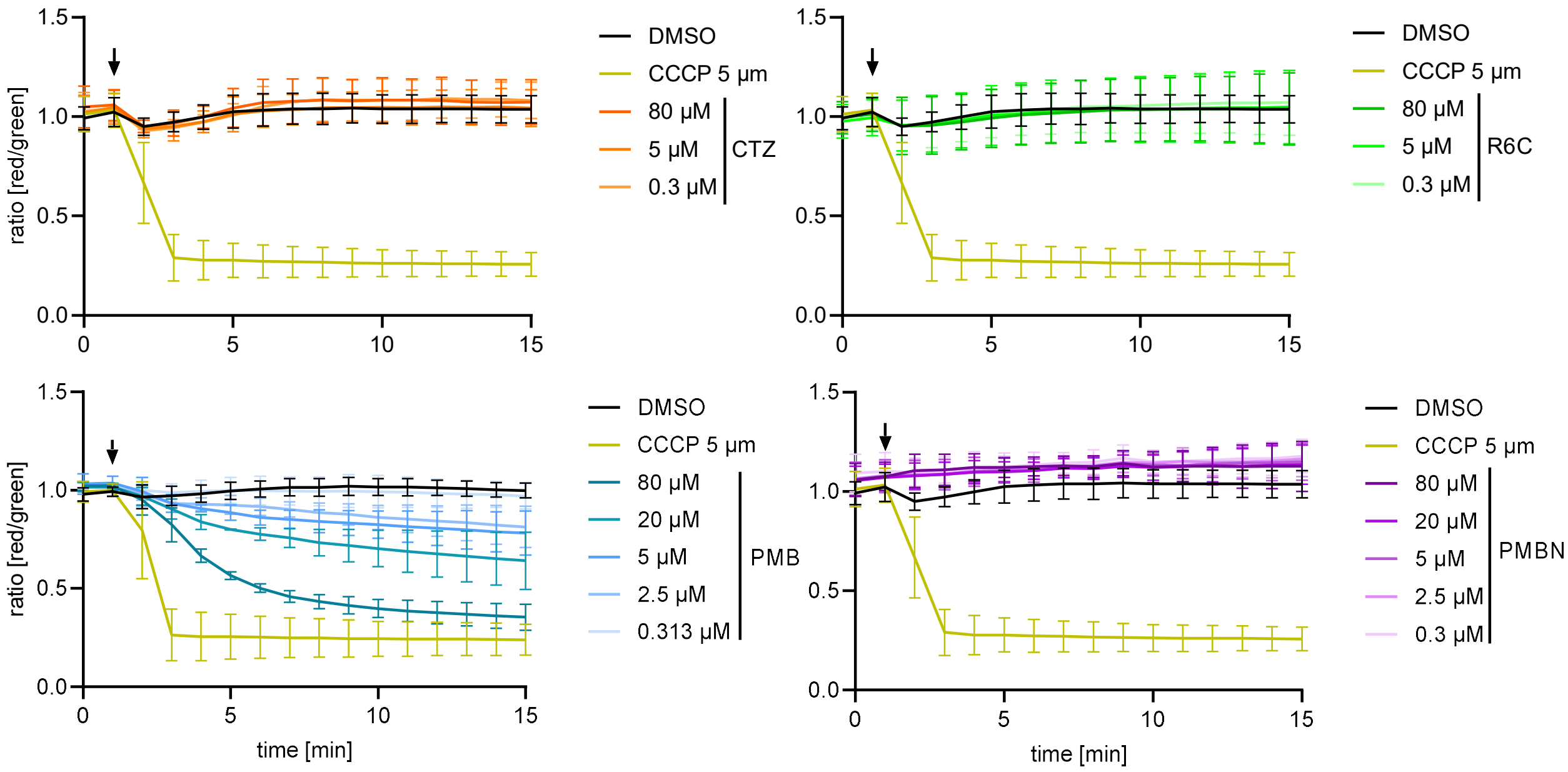

Figure S3. CTZ, R6C and polymyxin B nonapeptide (PMBN) showed no effect on the membrane potential in *B. subtilis* cells during 15 min of compound exposure, while concentration-dependent depolarisation was observed for polymyxin B (PMB) (n=3, ± SD). DMSO (1%) served as negative control, CCCP (5 µM) as positive control. The arrow marks compound addition.

Supplementary References

1. Bohorquez, L.C., et al., *The Conserved DNA Binding Protein WhiA Influences Chromosome Segregation in Bacillus subtilis.* J Bacteriol, 2018. **200**(8).
2. Ulijasz, A.T., A. Grenader, and B. Weisblum, *A vancomycin-inducible lacZ reporter system in Bacillus subtilis: induction by antibiotics that inhibit cell wall synthesis and by lysozyme.* J Bacteriol, 1996. **178**(21): p. 6305-9.
3. Piggot, P.J., *Mapping of asporogenous mutations of Bacillus subtilis: a minimum estimate of the number of sporeulation operons.* J Bacteriol, 1973. **114**(3): p. 1241-53.
4. Urban, A., et al., *Novel whole-cell antibiotic biosensors for compound discovery.* Appl Environ Microbiol, 2007. **73**(20): p. 6436-43.
5. Koo, B.M., et al., *Construction and Analysis of Two Genome-Scale Deletion Libraries for Bacillus subtilis.* Cell Syst, 2017. **4**(3): p. 291-305 e7.
6. Peters, J.M., et al., *A Comprehensive, CRISPR-based Functional Analysis of Essential Genes in Bacteria*. Cell, 2016. **165**(6): p. 1493-1506.
7. Fey, P.D., et al., *A genetic resource for rapid and comprehensive phenotype screening of nonessential Staphylococcus aureus genes.* mBio, 2013. **4**(1): p. e00537-12.
8. Peschel, A., et al., *Inactivation of the dlt operon in Staphylococcus aureus confers sensitivity to defensins, protegrins, and other antimicrobial peptides.* J Biol Chem, 1999. **274**(13): p. 8405-10.
9. Weidenmaier, C., et al., *Lack of wall teichoic acids in Staphylococcus aureus leads to reduced interactions with endothelial cells and to attenuated virulence in a rabbit model of endocarditis.* J Infect Dis, 2005. **191**(10): p. 1771-7.
10. Fedtke, I., et al., *A Staphylococcus aureus ypfP mutant with strongly reduced lipoteichoic acid (LTA) content: LTA governs bacterial surface properties and autolysin activity.* Mol Microbiol, 2007. **65**(4): p. 1078-91.
11. Ernst, C.M., et al., *Gain-of-Function Mutations in the Phospholipid Flippase MprF Confer Specific Daptomycin Resistance*. mBio, 2018. **9**(6).
12. Baba, T., et al., *Construction of Escherichia coli K-12 in-frame, single-gene knockout mutants: the Keio collection*. Mol Syst Biol, 2006. **2**: p. 2006 0008.
13. Bianchi, M., et al., *Fosfomycin Uptake in Escherichia coli Is Mediated by the Outer-Membrane Porins OmpF, OmpC, and LamB*. ACS Infect Dis, 2024. **10**(1): p. 127-137.
14. Tietgen, M., et al., *Impact of the colistin resistance gene mcr-1 on bacterial fitness.* Int J Antimicrob Agents, 2018. **51**(4): p. 554-561.
15. Tietgen, M., et al., *Corrigendum to 'Impact of the colistin resistance gene mcr-1 on bacterial fitness' [International Journal of Antimicrobial Agents 51/4 (2018) 554-561].* Int J Antimicrob Agents, 2019. **53**(6): p. 878.
16. *DOI 10.13145/bacdive12759.20240510.9. BacDive: Leibniz Institute, DSMZ-German Collection of Microorganisms and Cell Cultures GmbH.*
17. Cao, H., et al., *Comparative genome and transcriptome analysis reveals distinctive surface characteristics and unique physiological potentials of Pseudomonas aeruginosa ATCC 27853.* BMC Genomics, 2017. **18**(1): p. 459.
18. *KRINKO: Ergänzung zur Empfehlung der KRINKO „Hygienemaßnahmen bei Infektionen oder Besiedlung mit multiresistenten gram negativen Stäbchen“ (2012) im Zusammenhang mit der von EUCAST neu definierten Kategorie „I“ bei der Antibiotika-Resistenzbestimmung: Konsequenzen für die Definition von MRGN. Epid Bull 2019;9:82 – 83 | DOI 10.25646/5916.*
19. *The European Committee on Antimicrobial Susceptibility Testing. Breakpoint tables for interpretation of MICs and zone diameters. Version 16.0, 2026.* http://www.eucast.org.
20. *P.-E.-G.f.I.e.V., S2k-Leitlinie Kalkulierte parenterale Initialtherapie bakterieller Erkrankungen bei Erwachsenen. 01.12.2017, AWMF.*

21. Stachyra, T., et al., *Mechanistic studies of the inactivation of TEM-1 and P99 by NXL104, a novel non-beta-lactam beta-lactamase inhibitor*. Antimicrob Agents Chemother, 2010. **54**(12): p. 5132-8.
